# Phosphorylation of AMPA Receptors Is Required for Sensory Deprivation-Induced Homeostatic Synaptic Plasticity

**DOI:** 10.1371/journal.pone.0018264

**Published:** 2011-03-31

**Authors:** Anubhuti Goel, Linda W. Xu, Kevin P. Snyder, Lihua Song, Yamila Goenaga-Vazquez, Andrea Megill, Kogo Takamiya, Richard L. Huganir, Hey-Kyoung Lee

**Affiliations:** 1 Department of Biology, University of Maryland, College Park, Maryland, United States of America; 2 Neuroscience and Cognitive Science (NACS) Program, University of Maryland, College Park, Maryland, United States of America; 3 Cell Biology and Molecular Genetics (CBMG) Program, University of Maryland, College Park, Maryland, United States of America; 4 Department of Integrative Physiology, University of Miyazaki Faculty of Medicine, Miyazaki, Japan; 5 Solomon H. Snyder Department of Neuroscience, Johns Hopkins School of Medicine, Baltimore, Maryland, United States of America; University of Muenster, Germany

## Abstract

Sensory experience, and the lack thereof, can alter the function of excitatory synapses in the primary sensory cortices. Recent evidence suggests that changes in sensory experience can regulate the synaptic level of Ca^2+^-permeable AMPA receptors (CP-AMPARs). However, the molecular mechanisms underlying such a process have not been determined. We found that binocular visual deprivation, which is a well-established *in vivo* model to produce multiplicative synaptic scaling in visual cortex of juvenile rodents, is accompanied by an increase in the phosphorylation of AMPAR GluR1 (or GluA1) subunit at the serine 845 (S845) site and the appearance of CP-AMPARs at synapses. To address the role of GluR1-S845 in visual deprivation-induced homeostatic synaptic plasticity, we used mice lacking key phosphorylation sites on the GluR1 subunit. We found that mice specifically lacking the GluR1-S845 site (GluR1-S845A mutants), which is a substrate of cAMP-dependent kinase (PKA), show abnormal basal excitatory synaptic transmission and lack visual deprivation-induced homeostatic synaptic plasticity. We also found evidence that increasing GluR1-S845 phosphorylation alone is not sufficient to produce normal multiplicative synaptic scaling. Our study provides concrete evidence that a GluR1 dependent mechanism, especially S845 phosphorylation, is a necessary pre-requisite step for *in vivo* homeostatic synaptic plasticity.

## Introduction

Experience-dependent regulation of excitatory synaptic function is well documented in many brain areas. Recent studies highlight that experience alters the synaptic content of Ca^2+^-permeable AMPARs (CP-AMPARs) [1], [2], [3], [4], [5]. Most AMPARs on the principal neurons are impermeable to Ca^2+^, because they contain the GluR2 (or GluA2) subunit [6]. However, certain types of *in vivo* manipulations, such as sensory experience [2] or deprivation [4], drug exposure [1] or withdrawal [3], and ischemic insult [7], allow CP-AMPARs to be expressed at synapses. In some cases, like ischemic insult and cocaine exposure, regulation of CP-AMPARs is mediated by the GluR2 subunit via its interaction with Pick-1 [1], [7]. However, others, especially some models of homeostatic synaptic plasticity, are associated with alterations in the level of synaptic GluR1 with little change in GluR2 [4], [8], [9], [10], [11], [12], [13]. These results implicate GluR1-mediated mechanisms in the regulation of synaptic CP-AMPARs, but this is not without controversy. Under some conditions homeostatic synaptic scaling is associated with a co-regulation of GluR1 and GluR2 subunits [14], [15], or exclusively depends on GluR2 regulatory mechanisms [16]. The majority of what we know of the molecular mechanisms of synaptic scaling have come from *in vitro* culture systems, where neuronal activity is manipulated by pharmacological means. Therefore, it remains to be determined which mechanisms operate *in vivo* with sensory experience where activity changes may differ between brain areas and may be difficult to fully mimic by *in vitro* manipulations.

In the rodent primary visual cortex, a few days of binocular visual deprivation by dark-exposure leads to a global increase in excitatory synaptic transmission, which in juveniles follows the rules of multiplicative synaptic scaling [4], [17], [18]. Therefore, this is a useful *in vivo* model to elucidate the mechanisms of homeostatic synaptic plasticity. The binocular visual deprivation-induced homeostatic synaptic changes are accompanied by the appearance of CP-AMPARs and an increase in the GluR1 content at synapses [4], [18], which suggests synaptic incorporation of GluR1-homomers. Synaptic targeting of GluR1 has been linked to phosphorylation of several residues [reviewed in [19]]. For instance, GluR1-S845, a PKA site [20], and GluR1-S818, a protein kinase C (PKC) site [21], are both critical for targeting GluR1-containing AMPARs to synapses [21], [22]. In contrast, GluR1-S831, a site phosphorylated by Ca^2+^/calmodulin-dependent protein kinase II (CaMKII) and PKC [20], is not necessary for synaptic targeting [23], but increases single channel conductance [24], [25]. Reversible regulation of GluR1-S831 and GluR1-S845 phosphorylation correlates with bidirectional synapse-specific plasticity in the hippocampus [26], [27] and is required for spike-timing dependent plasticity in the visual cortex [28].

Here we examined whether phosphorylation of the GluR1 subunit is involved in synaptic trafficking of GluR1-homomers associated with *in vivo* homeostatic synaptic plasticity. We present evidence that homeostatic regulation of CP-AMPARs in the visual cortex depends on phosphorylation of GluR1 at the S845 site using gene knock-in mice lacking this site (GluR1-S845A mutants). However, increasing phosphorylation of the S845 site alone was not sufficient to produce normal multiplicative synaptic scaling. Our results suggest that the GluR1-S845 site is a necessary pre-requisite step for *in vivo* homeostatic synaptic scaling induced by sensory deprivation.

## Results

### Dark-exposure increases mEPSC amplitude and functional CP-AMPARs

**Figure 1:**
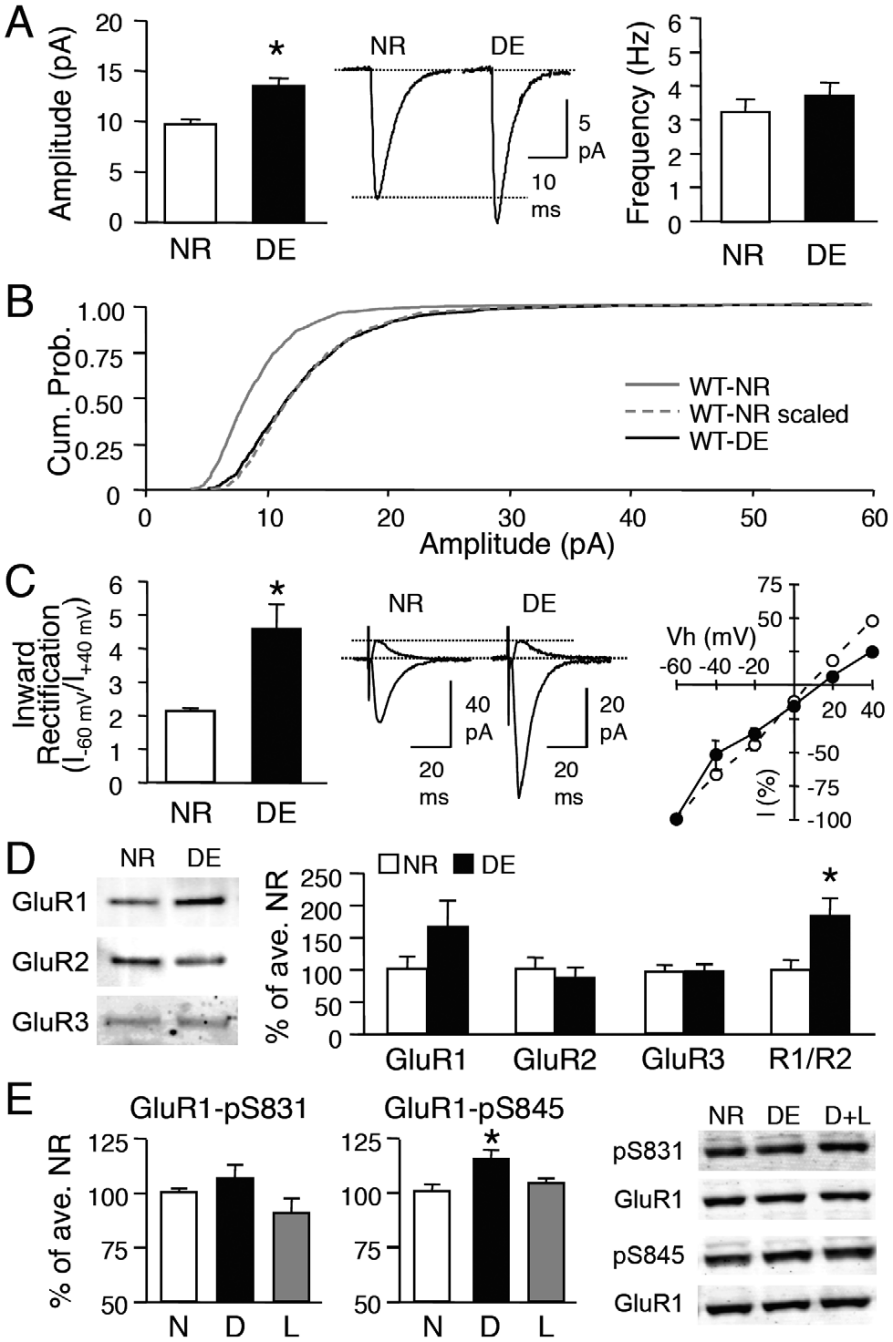
Visual deprivation induces homeostatic changes in excitatory synaptic transmission of layer 2/3 neurons in WT mice. **(A)** Left: Two days of DE (P21-P23) significantly increased the average mEPSC amplitude (*: t-test, p<0.001). Middle: Average mEPSC traces from WT-NR and WT-DE mice. Right: No difference in the average mEPSC frequency. **(B)** Cumulative probability of mEPSC amplitude of WT-DE (black solid line) is shifted to larger values (rightward shift) compared to WT-NR (gray solid line). When mEPSC amplitudes of WT-NR are multiplied by a factor (1.4) to match the average mEPSC amplitude of WT-DE, the cumulative probability curve (WT-NR scaled, gray dotted line) superimposes completely on the WT-DE curve (adjusted by removing the noise cut-off). This suggests that DE multiplicatively scales up mEPSC amplitudes. **(C)** Left: DE significantly increased the average inward rectification index (I_–60 mV_/I_+40 mV_) of evoked AMPAR-EPSC (*: t-test, p<0.02). Middle: Superimposed representative AMPAR-EPSC traces measured at -60 mV and +40 mV for NR and DE conditions. Right: I-V plot of evoked AMPAR-EPSC. Note that the I-V curve is linear in NR mice (open circles) and inward rectifying (black circles) in DE mice. **(D)** DE increased the GluR1/GluR2 (R1/R2) ratio in isolated PSD fractions from the visual cortex. Comparison of GluR1 (left), GluR2 (2^nd^ from left), and GluR3 (3^rd^ from left) levels and the R1/R2 ratio (rightmost) at the PSD of NR and DE. Left panel: Example immunoblots probed with antibody against GluR1 C-terminal, GluR2 N-terminal, and GluR3. *: t-test, p<0.02. **(E)** Left: No significant change in GluR1-S831 phosphorylation across NR (N), DE (D) and D+L (L) groups. Middle: DE (D) significantly increased GluR1-S845 phosphorylation compared to NR (N) and D+L (L). Right: Sample immunoblots probed with phospho-specific antibody to GluR1-S831 (pS831) and GluR1-S845 (pS845). Each blot was simultaneously probed with a GluR1 C-terminal antibody (GluR1). *: Significantly different from NR and D+L at p<0.001 with Fisher's PLSD posthoc test following a one-factor ANOVA.

Previous studies showed that 2 days of visual deprivation initiated at postnatal day 21 (P21) homeostatically scales up excitatory synaptic strength in layer 2/3 pyramidal neurons of the rodent visual cortex [17], [18], [29], hence we restricted our study to this age group. To ensure that wildtype (WT) mice with the same genetic background as the mutants show visual deprivation-induced homeostatic synaptic plasticity, we dark-exposed (DE) WT littermates of GluR1-S831A and GluR1-S845A mutants for 2 days. Normal-reared (NR) GluR1-S831 WT and GluR1-S845 WT showed no significant difference in the average AMPAR-mediated miniature excitatory postsynaptic current (mEPSC) amplitude, frequency, or kinetics (Table S1). Therefore, we combined the data obtained from the two WT lines, and compared mEPSCs between NR and DE groups. Consistent with previous reports [17], [18], [29], 2 days of DE shifted the distribution of mEPSC amplitude towards larger values, resulting in a significant increase in the average mEPSC amplitude (WT-NR: 9.7±0.4 pA, n = 19; WT-DE: 13.4±0.7 pA, n = 8; t-test, p<0.001; Fig. 1A). There was no significant change in the average mEPSC frequency (WT-NR: 3.2±0.4 Hz, n = 19; WT-DE: 3.7±0.4 Hz, n = 8; t-test, p>0.3; Fig. 1A) suggesting a postsynaptic locus of change. It has been demonstrated that homeostatic synaptic scaling occurs in a multiplicative manner, which is thought to preserve the relative difference in initial synaptic weight across synapses [30]. Consistent with our previous studies [17], [18], the DE-induced increase in mEPSC amplitude at this age followed the rules of “multiplicative scaling” (Fig. S1) with a scaling factor of 1.4 (Fig. 1B).

In addition to changes in mEPSC amplitude, AMPARs in layer 2/3 synapses of DE mice displayed inward rectification upon stimulation of layer 4 [inward rectification index (IR, I_–60 mV_/I_+40 mV_): WT-NR  = 2.1±0.1, n = 13; WT-DE  = 4.5±0.7, n = 6; t-test, p<0.02; Fig. 1C]. This suggests that DE increases functional CP-AMPARs at synapses. Consistent with this, we found an increase in the GluR1 to GluR2 (GluR1/GluR2) ratio of the postsynaptic density (PSD) fraction isolated from the visual cortex of DE compared to NR mice (WT-NR  = 100±12.4% of average WT-NR, n = 11; WT-DE  = 183±26.3%, n = 10; t-test, p<0.02; Fig. 1D), which was largely due to an increase in the GluR1 level. Our data are consistent with an interpretation that 2 days of DE changes the subunit composition of synaptic AMPARs, likely by recruiting Ca^2+^-permeable GluR1-homomers. This idea is further supported by our observation that there is a significant reduction in mEPSC decay time constant (τ) with 2 days of DE (Table S1), because GluR1-homomers exhibit a shorter decay time constant compared to GluR1/GluR2-heteromers [31]. In addition to changes in AMPAR subunit composition, DE caused a significant increase in the level of GluR1-S845 phosphorylation, which was readily reversed by 1 day of light exposure (WT-NR: 100±1.3% of average WT-NR, n = 8; WT-DE: 115±2.8%, n = 7; WT-D+L: 104±2.6%, n = 8; ANOVA: F(2,20) = 10.494, p<0.001; Fig. 1E). However, there was no significant change in GluR1-S831 phosphorylation (WT-NR: 100±1.6% of average WT-NR, n = 7; WT-DE: 107±5.8%, n = 9; WT-D+L: 91±6.3%, n = 7; ANOVA: F(2,20) = 2.332, p = 0.1; Fig. 1E). These results suggest that the regulation of the GluR1-S845 site may be responsible for recruiting CP-AMPARs at synapses following DE.

### GluR1-S845A mutant mice display larger mEPSC amplitude and contain CP-AMPARs under basal conditions

**Figure 2:**
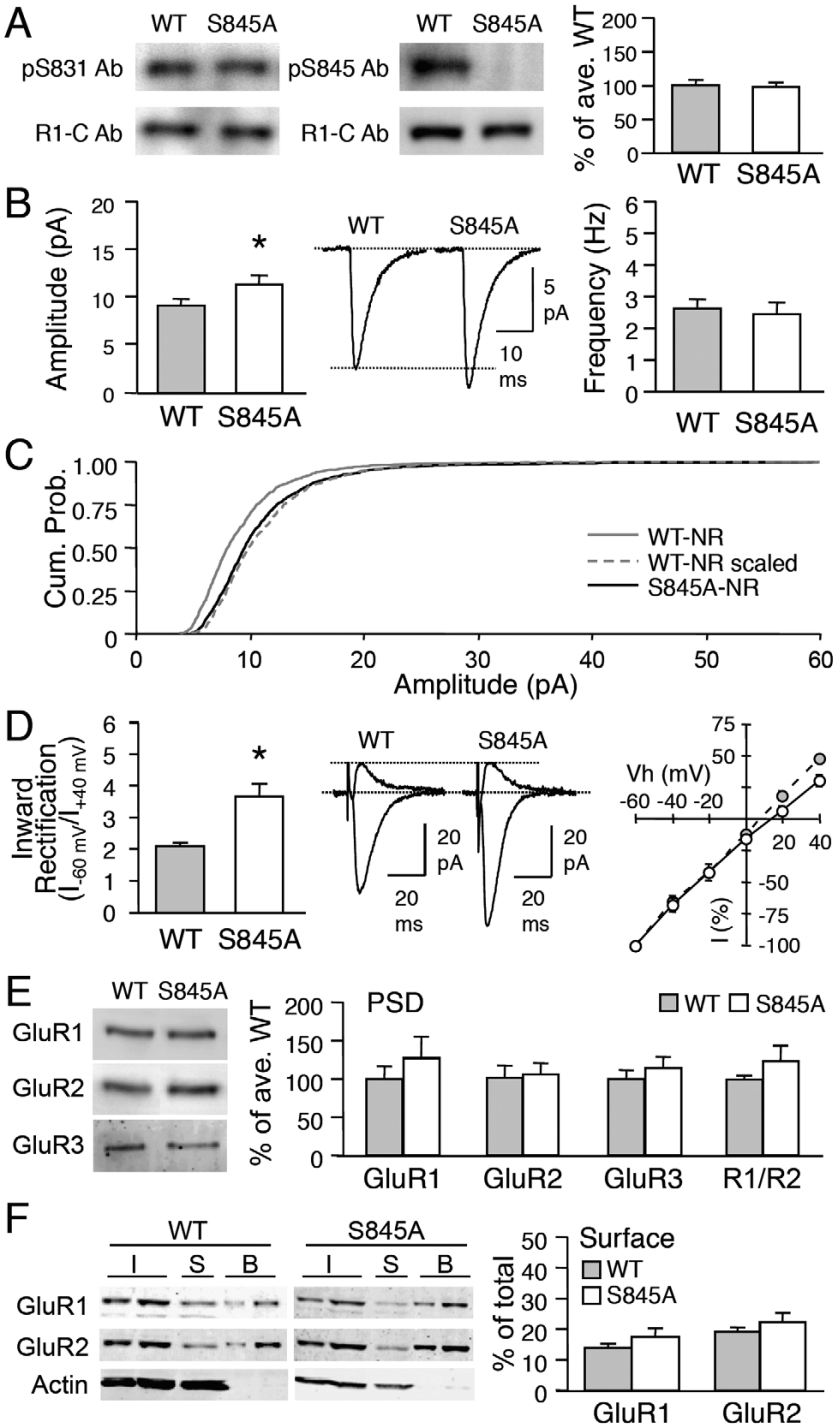
GluR1-S845A mutants have larger mEPSCs and have functional CP-AMPARs under basal conditions. **(A)** Immunoblot analysis of visual cortex samples from normal-reared WT and S845A mutants. Left: Sample immunoblot probed simultaneously with phospho-antibody for GluR1-S831 (pS831 Ab) and GluR1 C-terminal antibody (R1-C Ab). Middle: Sample immunoblot simultaneously probed with phospho-antibody for GluR1-S845 (pS845 Ab) and R1-C Ab. Note the absence of pS845 Ab signal in S845A sample. Right: Quantification of relative phosphorylation at GluR1-S831 in WT and S845A mutants. **(B)** Left: Significantly larger average basal mEPSC amplitude in GluR1-S845A mutants (*: t-test, p<0.05). Middle: Average mEPSC traces from WT and S845A. Right: No change in average mEPSC frequency. **(C)** GluR1-S845A mutants display larger mEPSC amplitude values under basal conditions (normal-reared) when compared to WTs. This is shown as a rightward shift in the cumulative probability graph of S845A-NR (black solid line) when compared to that of WT-NR (gray solid line). The amplitude of individual mEPSCs recorded from WT-NR was multiplied by a factor (1.2) to allow the average mEPSC amplitude of WT NR to match that of S845A-NR (WT-NR scaled, gray dotted line). The cumulative probability curve of WT-NR scaled (gray dotted line) was statistically significantly different from the S845A-NR curve (black solid line) (p<0.05, Kolmogrov-Smirnov test) suggesting that S845A mutation does not multiplicatively scale up mEPSCs compared to WT. **(D)** Left: Significantly larger inward rectification index of evoked AMPAR-EPSC from S845A mutants (*: t-test, p<0.01). Middle: Superimposed inward (Vh = –60 mV) and outward (Vh = +40 mV) currents through AMPARs from NR WT and S845A. Right: I-V plot of evoked AMPAR-EPSC. Note: I-V curve of S845A mice is inward rectifying (open circles) compared to WTs (gray circles). **(E)** No difference in GluR1 (left), GluR2 (2nd from left), GluR3 (3^rd^ from left), and GluR1/GluR2 (R1/R2) ratio (right) in PSDs of WT and GluR1-S845A. Left panel: Example blots. **(F)** Left: Example immunoblots of steady-state biotinylation on isolated layer 2/3 visual cortex slices from wildtype (WT) and S845A mutant (S845A). Different amount of total sample (I, input: 2.5% and 5% of total input each lane), intracellular fraction (S, supernatant), and surface biotinylated fraction (B: 10% and 20% of total biotinylated sample each lane) were loaded to the gel, and probed for GluR1, GluR2, and actin. Right: Quantification of surface GluR1 and GluR2 expressed as a percentage of total GluR1 and GluR2 from each blot. No significant difference in GluR1 or GluR2 was observed between wildtypes and S845A mutants.

To determine whether GluR1-S845 phosphorylation is necessary for increasing mEPSC amplitude and synaptic incorporation of CP-AMPARs with DE, we used GluR1-S845A mutants [28], [32], [33]. As expected, visual cortex samples from GluR1-S845A mutants lacked a signal when probed with a phospho-specific antibody to S845 (Fig. 2A). There was no significant change in the S831 phosphorylation in the GluR1-S845A mutants compared to the WT littermates (WT: 100±5% of average WT, n = 14; S845A: 145±31% of average WT, n = 12; t-test, p = 0.16; Fig. 2A).

To investigate whether lacking GluR1-S845 alters basal synaptic function, we compared AMPAR-mediated mEPSCs recorded from layer 2/3 pyramidal neurons from age-matched (P21-P23) NR GluR1-S845A and WT littermates. GluR1-S845A mutants showed significantly larger basal mEPSC amplitude in comparison to their WT counterparts (WT: 9.22±0.55 pA, n = 11; S845A: 11.4±0.9 pA, n = 10; t-test, p<0.05; Fig. 2B), without changes in mEPSC frequency (WT: 2.6±0.2 Hz, n = 11; S845A: 2.4±0.3 Hz, n = 10; t-test p>0.6; Fig. 2B) or kinetics (Table S1). The mEPSC amplitude distribution shifted towards larger values in the GluR1-S845A mutants, but was not quite multiplicative when compared to WTs (Kolmogrov-Smirnov test, p<0.05: Fig. 2C).

To determine whether the larger basal mEPSC amplitude in the GluR1-S845A mutants is accompanied by a change in the subunit composition of synaptic AMPARs, we compared the current-voltage (I-V) relationship of evoked AMPAR-EPSCs of GluR1-S845A mutants and their WT littermates. We found significantly larger average inward rectification index from GluR1-S845A mutants (WT: 2.1±0.10, n = 8; S845A: 3.7±0.38, n = 10; t-test, p<0.01; Fig. 2D), which was dependent on the presence of intracellular spermine (Fig. S2A). Surprisingly, the change in the I-V curve of evoked AMPAR current was not accompanied by an alteration in GluR1 and GluR2 amount or the GluR1/GluR2 ratio of the PSD (GluR1: WT = 100±15%, n = 7, S845A  = 127±27%, n = 8, t-test, p>0.4; GluR2: WT = 100±15%, n  = 7, S845A  = 104±14%, n = 8, t-test, p>0.8; Fig. 2E). Neither was there a significant change in the GluR1/GluR2 ratio between wildtype and S845A mutants in the synaptic plasma membrane (SPM) fraction (WT = 100±8.2%, n = 7; S845A  = 110±17.6%, n = 7; t-test: p>0.6). Next, we examined whether there is an up-regulation of GluR1 homomers in the extrasynaptic plasma membrane of GluR1-S845A mutants by performing steady-state biotinylation of visual cortical slices. Since the changes could be restricted to layer 2/3, we cut the slices to remove other layers. Using this method, we found no significant change in the surface expression of GluR1 or GluR2 between wildtypes and GluR1-S845A mutants (surface GluR1: WT = 14±1.2% of total, n = 8 mice; S845A  = 17±2.8% of total, n = 9 mice, t-test, p>0.2; surface GluR2: WT = 19±1.4% of total, n = 8 mice; S845A  = 22±2.9% of total, n = 9 mice, t-test, p>0.3; Fig. 2F). These results suggest that the enhancement of functional CP-AMPARs at layer 2/3 synapses of GluR1-S845A mutants are not associated with an up-regulation of extrasynaptic plasma membrane pool of GluR1 homomers.

### Visual deprivation-induced homeostatic synaptic plasticity is absent in GluR1-S845A mutants

**Figure 3:**
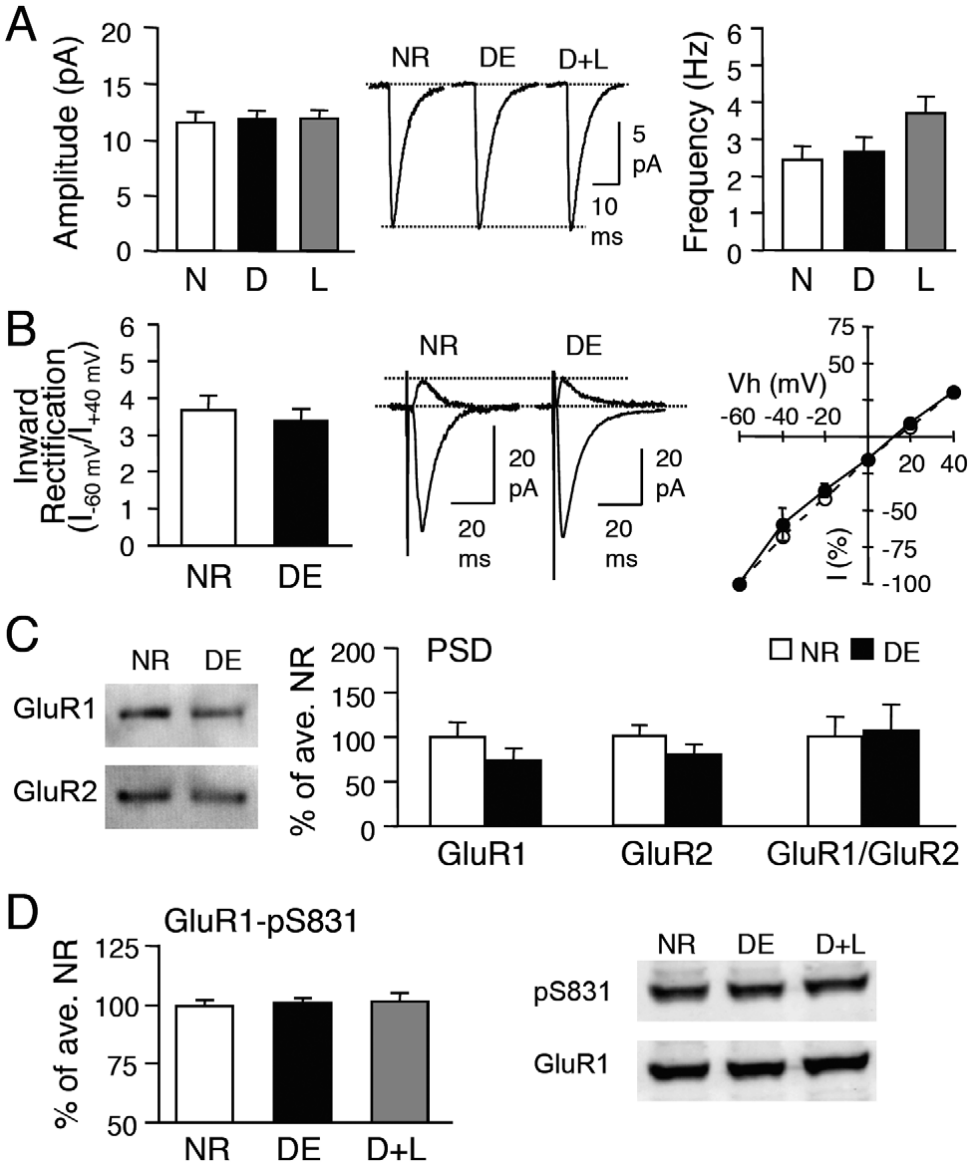
Visual experience-induced homeostatic synaptic changes are absent in GluR1-S845A mutants. **(A)** No significant change in average mEPSC amplitude (left) or frequency (right) across NR (normal-reared until P23), DE (dark-exposed for 2 days from P21-P23), and D+L (2 days DE followed by 1 day of light exposure) groups of GluR1-S845A mutants. Middle: Average mEPSC traces from each group. **(B)** No change in inward rectification index between NR and DE GluR1-S845A mutants. **(C)** DE did not alter GluR1 or GluR2 levels in the PSD of GluR1-S845A mutants. **(D)** No alterations in GluR1-S831 phosphorylation in the visual cortex of GluR1-S845A mutants.

The increase in mEPSC amplitude of visual cortical neurons by 2 days of DE is rapidly reversed by 1 day of light exposure [17], [18]. To investigate the role of GluR1-S845 in the reversible regulation of synaptic transmission, we dark-exposed GluR1-S845A mutants (from P21 to P23) and re-exposed them to light for 1 day. Unlike in WT mice, DE or a subsequent light exposure (D+L), failed to alter the average mEPSC amplitude in the GluR1-S845A mutants (S845A-NR: 11.4±0.9 pA, n = 10; S845A-DE: 11.7±0.6 pA, n = 11; S845A-D+L: 11.8±0.7 pA, n = 12; ANOVA: F (2,30) = 0.067, p>0.9; Fig. 3A). There was also no significant change in mEPSC frequency across the 3 groups (S845A-NR: 2.4±0.3 Hz, n = 10; S845A-DE: 2.7±0.4 Hz, n = 11; S845A-D+L: 3.7±0.4 Hz, n = 12; ANOVA: F (2,30) = 3.194, p>0.05; Fig. 3A).

Furthermore, 2 days of DE did not modify the AMPAR subunit composition at the synapses of GluR1-S845A mutants, as there was no significant change in the inward rectification index (S845A-NR: 3.7±0.38, n = 10; S845A-DE: 3.4±0.31, n = 6; t-test, p>0.5; Fig. 3B). Neither was there a significant change in the level of GluR1 or GluR2 at the PSD (GluR1: S845A-NR: 100±16%, n = 7; S845A-DE: 73±13%, n = 8; t-test, p>0.2; GluR2: S845A-NR: 100±11%, n = 7; S845A-DE: 79±11%, n = 8; t-test, p>0.1; Fig. 3C). As expected, manipulation of visual experience did not alter the phosphorylation level of the GluR1-S831 site (S845A-NR: 100±2.2% of average NR, n = 11; S845A-DE: 101±2.0%, n = 6; S845A-D+L: 102±3.6, n = 6; ANOVA: F(2,20) = 0.127, p = 0.88; Fig. 3D). These results suggest that mutation of the GluR1-S845 phosphorylation site prevents visual experience-induced homeostatic changes in synaptic strength.

### GluR1-S845 phosphorylation increases the amplitude of mEPSCs but not via multiplicative scaling

**Figure 4:**
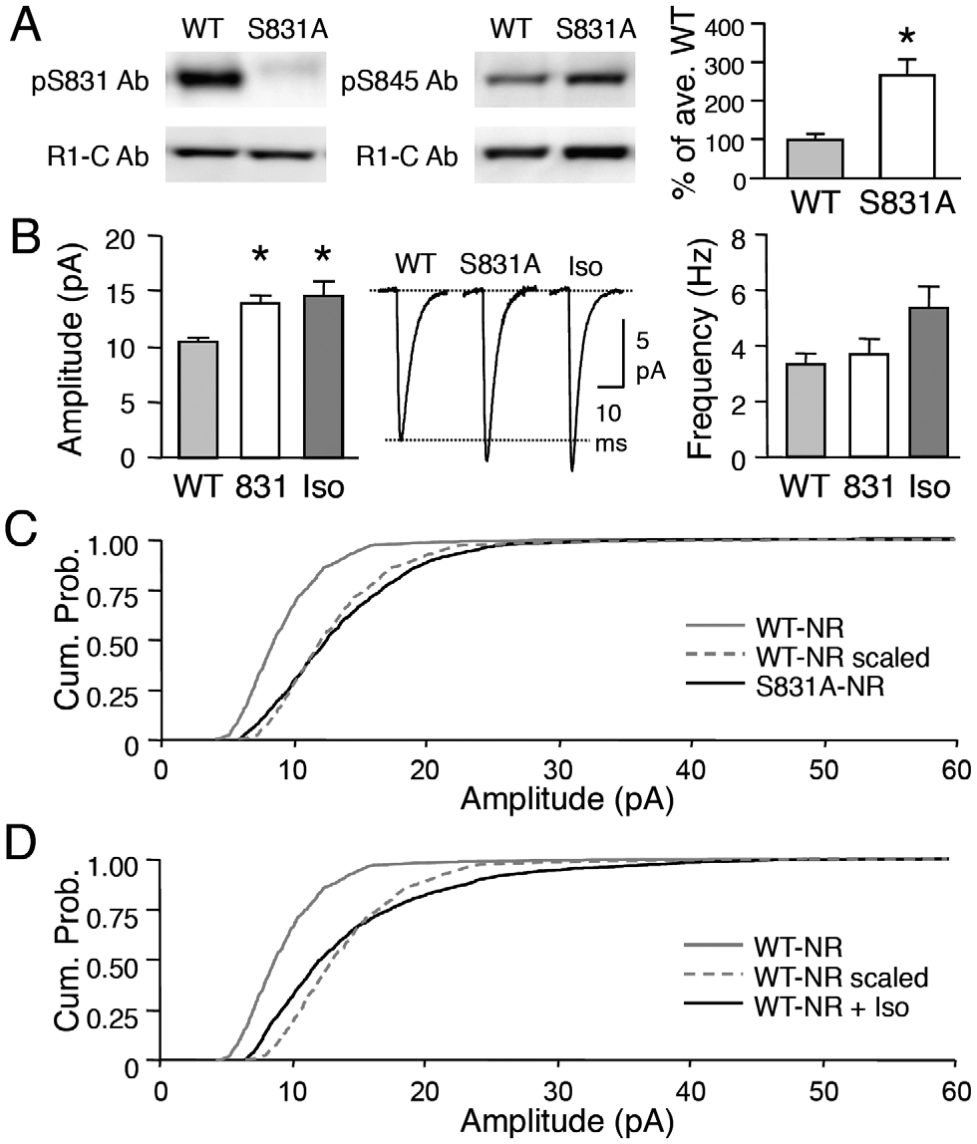
Abnormally enhanced GluR1-S845 phosphorylation and synaptic transmission in GluR1-S831A mutants. **(A)** Left: Example immunoblots of WT and GluR1-S831A mutant visual cortex samples. Note the lack of phosphorylated S831 (pS831) signal (upper left), while normal expression of GluR1 (as measured with GluR1-C terminal Ab, bottom left blot). S831A mutants display a significant increase in the remaining GluR1-S845 phosphorylation (example blots in the middle panel, quantification in the right graph). *: p<0.01, t-test. **(B)** Average mEPSC amplitude is increased in S831A mutants (labeled 831) as well as in WT visual cortex slices treated with isoproterenol (Iso). Middle panel: average mEPSC traces from each group. Right panel: Isoproterenol treated group showed a trend of an increase in mEPSC frequency, which did not reach statistical significance (p>0.05, one-factor ANOVA). *: Significantly different from WT at p<0.01 with Fisher's PLSD posthoc test after a one-factor ANOVA. **(C)** GluR1-S831A mutation did not cause multiplicative scaling of mEPSCs. The amplitude of mEPSCs of S831A-NR (black solid line) shifted to larger values compared to WT-NR (gray solid line). Amplitudes of individual mEPSCs recorded from WT-NR were multiplied by a scaling factor (1.4) to match the average mEPSC amplitude to that of S831A-NR to generate the WT-NR scaled (gray dotted line). However, the cumulative probability curve of WT-NR scaled did not superimpose that of S831A-NR (p<0.01, Kolmogrov-Smirnov test). This suggests that the increase in mEPSC amplitude in GluR1-S831A mutants is not due to multiplicative scaling. **(D)** Treating WT-NR visual cortex slices with isoproterenol (WT-NR+iso, black solid line) increased mEPSC amplitude as shown by a rightward shift in the curve compared to control WT-NR (gray solid line). We multiplied the amplitude of mEPSCs from WT-NR with a scaling factor (1.5) to match the average mEPSC amplitude of isoproterenol treated group to generate the WT-NR scaled (gray dotted line). The cumulative probability curve of the WT-NR scaled did not overlap with the WT-NR+Iso group (p<0.01, Kolmogrov-Smirnov test).

While our data suggest that GluR1-S845 is necessary for visual deprivation-induced homeostatic synaptic plasticity, we found that phosphorylation of this site may not be sufficient. The first line of evidence came from our study of another mutant mouse line specifically lacking the GluR1-S831 site (GluR1-S831A mutant). Biochemical characterization of visual cortex samples from GluR1-S831A mutants revealed that the remaining GluR1-S845 site is highly phosphorylated under basal conditions compared to the WT littermates (WT: 100±15% of average WT, n = 10; S831A: 265±40% of average WT, n = 11; t-test: p<0.01, Fig. 4A). Therefore, we decided to use the GluR1-S831A mutants to test whether increasing GluR1-S845 phosphorylation is sufficient to scale up synapses in the visual cortex. We found that GluR1-S831A mutants show significantly larger average basal mEPSC amplitude compared to their WT counterparts (WT: 10.4±0.3 pA, n = 8; S831A: 13.8±0.7 pA, n = 11; t-test, p<0.001; Fig. 4B), without alterations in average mEPSC frequency (WT: 3.3±0.4 Hz, n = 8; S831A: 3.6±0.5 Hz, n = 11; t-test, p>0.6; Fig. 4B). However, the larger average mEPSC amplitude in GluR1-S831A mutants was not multiplicative (Fig. 4C).

**Figure 5:**
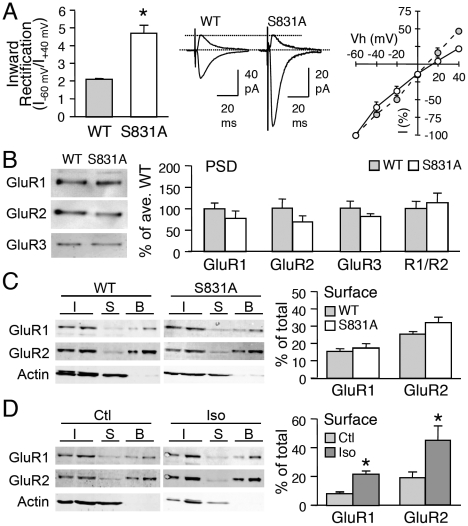
Comparison of AMPAR regulation in GluR1-S831A mutants and wildtypes treated with isoproterenol. **(A)** S831A mutants show increased inward rectification of AMPAR-EPSC evoked upon stimulation of layer 4. Middle: representative traces. Right: I-V curve of WT (gray circles) and S831A (open circles). *: p<0.001, t-test. **(B)** No changes in GluR1 (left), GluR2 (2^nd^ from left), GluR3 (3^rd^ from left) or GluR1/GluR2 (R1/R2) ratio (right) in isolated PSD fractions of WT and S831A visual cortex. Left panel: Representative blots. **(C)** Left: Example immunoblots of steady-state biotinylation in isolated layer 2/3 visual cortex slices from wildtype (WT) and S831A mutant (S831A). Different amount of total sample (I, input: 2.5% and 5% of total input each lane), intracellular fraction (S, supernatant), and surface biotinylated fraction (B: 10% and 20% of total biotinylated sample each lane) were loaded to the gel, and probed for GluR1, GluR2, and actin. Right: Quantification of surface GluR1 and GluR2 expressed as a percentage of total GluR1 and GluR2 from each blot. No significant difference in GluR1 or GluR2 was observed between wildtypes and S831A mutants. **(D)** Left: Example immunoblots of steady-state biotinylation of control (Ctl) and isoproterenol (Iso) treated isolated layer 2/3 visual cortex slices from wildtype. Right: Isoproterenol treatment significantly increased both GluR1 and GluR2 surface levels.

The basal changes in mEPSCs in GluR1-S831A mutants were strikingly similar to what we observed in the GluR1-S845A mutants. Furthermore, like the GluR1-S845A mutants, GluR1-S831A mutants showed significantly shorter mEPSC decay kinetics (Table S1), a larger inward rectification of AMPAR current (WT = 2.1±0.04, n = 5; S831A  = 4.7±0.43, n = 9; t-test, p<0.001; Fig. 5A), which was dependent on intracellular spermine (Fig. S2B), as well as no significant differences in GluR1 or GluR2 content at the PSDs (GluR1: WT = 100±13%, n = 8; S831A  = 78±16%, n = 8; t-test, p>0.3; GluR2: WT = 100±21%, n = 8; S831A  = 69±13%, n = 8; t-test, p>0.2; Fig. 5B). Neither was there a change in the cell surface expression of AMPARs measured by steady-state biotinylation of isolated superficial layers of visual cortical slices (surface GluR1: WT = 15±1.4% of total, n = 9 mice, S831A  = 17±2.4% of total, n = 10 mice, t-test: p>0.5; surface GluR2: WT = 25±1.3% of total, n = 9 mice, S831A  = 32±2.9% of total, n = 9 mice; t-test: p>0.06; Fig. 5C).

To further confirm whether the larger mEPSC amplitude in the GluR1-S831A mutants is due to the hyperphosphorylation of the GluR1-S845 site, we pharmacologically increased GluR1-S845 phosphorylation in WTs. We previously showed that a transient application of isoproterenol (a β-adrenergic receptor agonist) to visual cortical slices greatly and persistently (at least for 1 hour) increases GluR1-S845 phosphorylation without significant effects on the GluR1-S831 site [28]. Treating visual cortex slices with isoproterenol (5 µM with 10 µM ascorbic acid, 10 min) significantly increased the average amplitude of mEPSCs compared to WT (WT: 10.4±0.3 pA, n = 8; Iso: 14.4±1.3 pA, n = 9; t-test, p<0.02; Fig. 4B). However, the increase was not multiplicative (Fig. 4D) similar to what was seen in the GluR1-S831A mutants. However, in contrast to the GluR1-S831A mutants, isoproterenol treatment increased both GluR1 and GluR2 levels on the plasma membrane of isolated layer 2/3 slices from wildtype mice (surface GluR1: Control  = 9±0.8% of total, n = 7 mice, Iso  = 22±1.8% of total, n = 8 mice, t-test: p<0.001; surface GluR2: Control  = 20±3.5% of total, n = 7 mice, Iso  = 45±10.3% of total, n = 8 mice; t-test: p = 0.05; Fig. 5D), which occurred without alterations in the ratio of GluR1 and GluR2 at the surface (surface GluR1/GluR2 ratio: Control  = 0.57±0.089, Iso  = 0.66±0.123; t-test: p>0.5). Taken together with the lack of change in cell surface AMPARs of GluR1-S831A mutants, our results suggest that isoproterenol may recruit signals in addition to GluR1-S845 phosphorylation for up-regulating cell surface levels of both GluR1 and GluR2.

### Abnormal visual deprivation-induced regulation of AMPAR function in GluR1-S831A mutants

**Figure 6:**
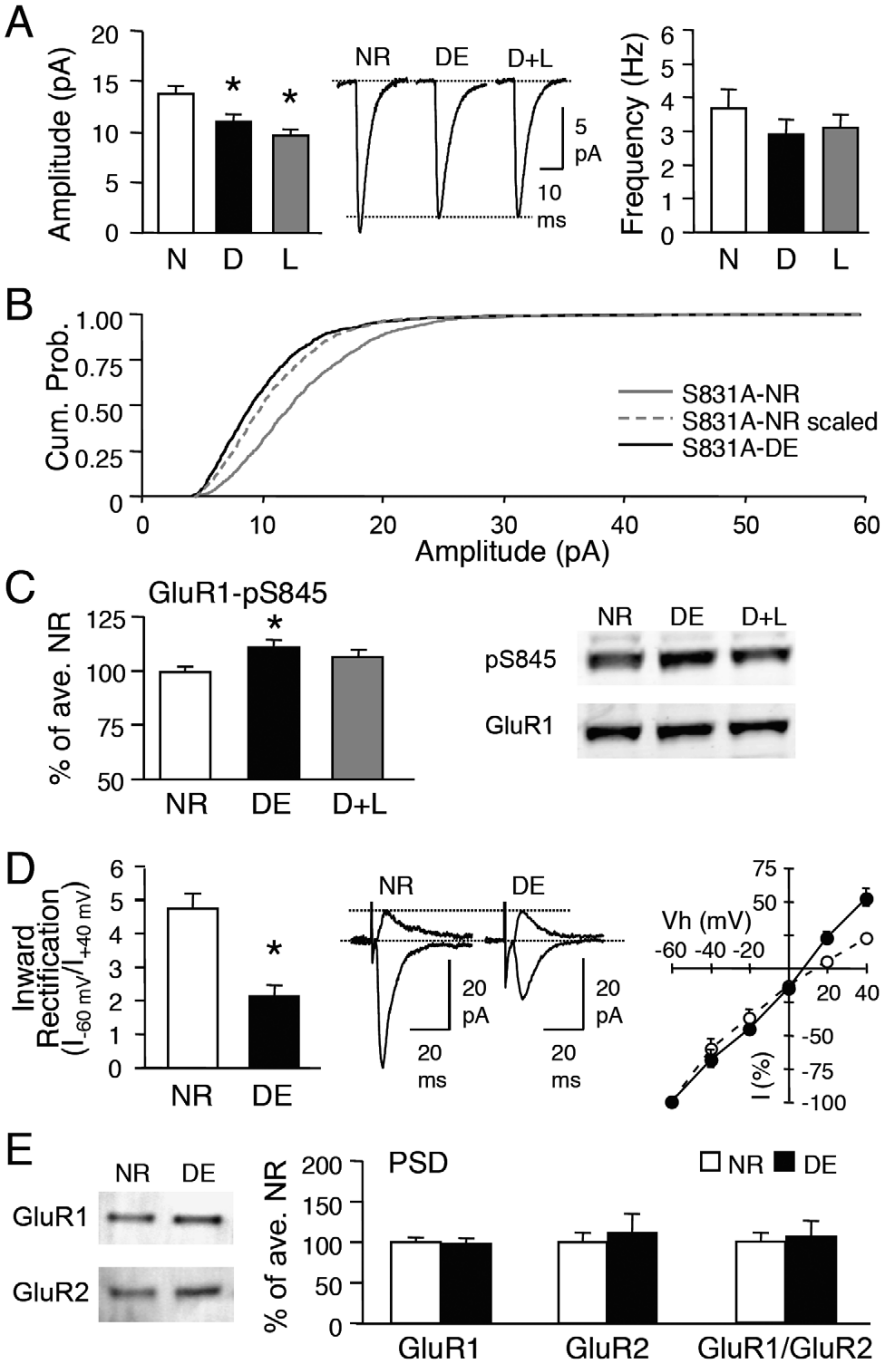
Abnormal visual experience-induced homeostatic synaptic plasticity in GluR1-S831A mutants. **(A)** Left: average mEPSC amplitude decreased with dark-exposure (D) and did not decrease any further with re-exposure to light (L). *: Significantly different from NR (N) at p<0.01, Fisher's PLSD posthoc test. Middle: average mEPSC traces from each group. Right: No significant changes in mEPSC frequency across groups. **(B)** Dark-exposed GluR1-S831A mutants displayed smaller mEPSC amplitudes as seen as a leftward shift in the cumulative probability curve of S831A-DE (black solid line) compared to S831A-NR (gray solid line). To determine whether this is due to multiplicative scaling down of mEPSCs, we multiplied the mEPSC amplitude of S831A-NR with a scaling factor (0.8) to match the average mEPSC amplitude of S831A-DE (S831A-NR scaled, gray dotted line). The cumulative probability curve of S831A-NR scaled did not match that of S831A-DE (p<0.01, Kolmogrov-Smirnov test) suggesting that the decrease in mEPSC size does not follow the rules of multiplicative scaling. **(C)** Left: GluR1-S831A mutants showed a normal increase in the remaining GluR1-S845 phosphorylation with DE. *: p<0.01, Fisher's PLSD posthoc test. Right: example immunoblots probed simultaneously with pS845 antibody (upper) and GluR1 C-terminal antibody (lower). **(D)** Left: dark-exposure (DE) GluR1-S831A mutants significantly reduced the inward rectification index. *: p<0.001, t-test. Middle: superimposed representative traces of evoked AMPAR-EPSC for NR and DE taken at −60 mV and +40 mV. Right: DE S831A mutants (black circles) show a more linear I-V curve than NR S831A mutants (open circles). **(E)** No change in GluR1 (left), GluR2 (middle), or GluR1/GluR2 ratio (right) in the PSD samples of NR and DE S831A mutants.

Next, we investigated whether visual deprivation-induced homeostatic synaptic plasticity is affected in the GluR1-S831A mutants. Surprisingly, GluR1-S831A mutants significantly decreased the average mEPSC amplitude when dark-exposed, and did not show any further decrease with light exposure (S831A-NR: 13.8±0.7 pA, n = 11; S831A-DE: 11.02±0.7 pA, n = 10; S831A-D+L: 9.6±0.6 pA, n = 9; ANOVA: F (2,27) = 10.995, p<0.001; Fig. 6A). There was no significant change in mEPSC frequency (S831A-NR: 3.6±0.5 Hz, n = 11; S831A-DE: 2.9±0.4 Hz, n = 10; S831A-D+L: 3.1±0.4 Hz, n = 9; ANOVA: F (2,27) = 0.801, p>0.4; Fig. 6A) or kinetics (Table S1) across the three conditions. While the distribution of mEPSC amplitude shifted towards smaller values in DE GluR1-S831A mutants, it did not follow the rules of “multiplicative scaling” (Fig. 6B). The reduction of mEPSC amplitude in DE GluR1-S831A mutants was not due to an abnormal regulation of the GluR1-S845 site, because S845 phosphorylation still increased when GluR1-S831A mutants were dark-exposed (S831A-NR: 100±2.1% of average NR, n = 6; S831A-DE: 110±2.7, n = 7; S831A-D+L: 106±3.1, n = 6; ANOVA: F(2,16) = 4.544, p<0.03; Fig. 6C).

In the course of analyzing the GluR1-S831A mutants, we noticed that the mEPSC decay kinetics became longer with DE (Table S1). This suggests that the reduction in mEPSC amplitude with DE may accompany a change in AMPAR subunit composition. To test this, we compared the I-V relationship of AMPAR-EPSC evoked by layer 4 stimulation. DE caused a significant decrease in the inward rectification index (S831A-NR: 4.7±0.43, n = 9; S831A-DR  = 2.1±0.31, n = 6; t-test, p<0.001) as apparent from a more linear I-V curve (Fig. 6D). This suggests that the decrease in mEPSC amplitude is accompanied by a loss of functional CP-AMPARs from the synapses. However, we did not find significant changes in GluR1 or GluR2 levels in the PSD of DE GluR1-S831A mutants (GluR1: S831A-NR: 100±5%, n = 9, S831A-DE: 98±7%, n = 8, t-test, p>0.7; GluR2: S831A-NR: 100±11%, n = 9. S831A-DE: 111±23%, n  = 8, t-test, p>0.6; Fig. 6E). Collectively, these results indicate that GluR1-S831A mutants undergo aberrant regulation of AMPARs with visual deprivation.

## Discussion

We demonstrated that *in vivo* homeostatic regulation of excitatory synapses in the superficial layers of the mouse visual cortex is dependent on the GluR1-S845 site. Visual deprivation-induced scaling up of mEPSC amplitude was associated with an increase in GluR1-S845 phosphorylation, and was absent in the GluR1-S845A mutants. The increase in mEPSC size by visual deprivation accompanied synaptic incorporation of Ca^2+^-permeable GluR1-homomers. Therefore the action of GluR1-S845 is likely at the level of regulating GluR1-homomers. While GluR1-S845 is necessary for the sensory deprivation-induced homeostatic synaptic plasticity, we have converging evidence that its phosphorylation may not be sufficient to mediate normal multiplicative scaling. This suggests that multiplicative scaling requires additional mechanisms beside GluR1-S845 phosphorylation. Collectively, our results suggest that phosphorylation at S845 on GluR1 is one of the critical determinants of *in vivo* homeostatic synaptic plasticity.

Our data are consistent with our previous findings [4] and the majority of *in vitro* scaling studies showing GluR1 regulation [8], [9], [10], [11], [12], [13], [34], and provides evidence that *in vivo* sensory deprivation-induced homeostatic synaptic changes require GluR1 phosphorylation. The role of GluR1-S845 phosphorylation is likely at the level of regulating the function of Ca^2+^-permeable GluR1-homomers, because it is correlated with the appearance of inward rectifying AMPARs. A recent study reported that visual deprivation-induced synaptic scaling does not recruit CP-AMPARs to synapses using monocular TTX injection as a way to deprive vision [16]. Dark-exposure and intraocular TTX injection are likely to affect retinal and thalamic (i.e. lateral geniculate nucleus, LGN) activity differently. For instance, intraocular TTX injection will suppress spontaneous retinal activity, as well as visually evoked activity, and is known to produce high frequency oscillatory activity in the LGN [35]. On the other hand, dark-exposure will not affect spontaneous retinal activity, and its effect on LGN activity is currently unknown. Whether this explains the discrepancy in mechanism for synaptic scaling in the visual cortex remains to be tested.

Unexpectedly, both GluR1-S831A and GluR1-S845A mutants showed larger basal mEPSC amplitude and inward rectification of AMPAR current. This is quite puzzling because the former mutant is hyperphosphorylated on the S845 site, while the latter lacks the site. The increase in basal CP-AMPARs in these mutants was not due to a compensatory increase in GluR3 (or GluA3)-containing AMPARs (Fig. 2E, 5B). One simple hypothetical scenario is that the GluR1-S845A mutation acts similar to phosphorylation of the serine residue, which is in line with the interpretation of results of an equivalent phosphorylation site on the GluR4 (or GluA4) subunit (GluR4-S842) [22]. In the GluR4 study, mimicking phosphorylation (S842D mutation) or preventing phosphorylation (S842A mutation) of the S842 site enhanced synaptic expression of the receptor [22]. Another surprise is that none of the mutants showed a change in the GluR1 content of the PSD, the SPM, or the plasma membrane fractions, which seemingly contradicts the appearance of functional CP-AMPARs. We surmise that the changes in basal AMPAR function in the phosphomutants may be by lateral movement of pre-existing surface receptors, likely perisynaptic receptors, into synapses, but that the mobilized receptors are not anchored to the PSD. Recent single molecule tracking studies reported that GluR1-containing AMPA receptors freely diffuse in and out of synapses even within the PSD area [36], [37], [38], which may represent the unanchored GluR1-homomer population. Our interpretation raises an interesting possibility that the role of the GluR1-S845 site is to regulate mobilization of AMPARs, but it does not control the movement *per se* or the stabilization of receptors at the synapses. It is likely that targeted movement of AMPARs to synapses will depend on small GTPases and myosin motors [39], [40], while anchoring to synapses may require PDZ interactions [23]. There are alternative possibilities for our observations. For instance, the changes in synaptic GluR1 content in the mutants may be restricted to layer 2/3, hence undetectable in the PSD or the SPM samples which are prepared from the whole visual cortex due to the requirement of a large starting material for effective subcellular fractionation. Also, considering that even addition of a small number of CP-AMPARs is predicted to alter synaptic transmission [41], the changes may be below the limit of biochemical detection. However, this interpretation is at odds with the measured changes in the GluR1/GluR2 ratio at the PSD following dark-exposure, unless the DE effect is more global. Another alternative is that the changes in AMPAR function may be due to altered single channel properties of pre-existing synaptic receptors. GluR1-S845 phosphorylation increases the mean open probability of the channel [42], hence could explain the increase in mEPSC amplitude in GluR1-S831A mutants. Further, if the S845 phosphorylation occurs on pre-existing synaptic GluR1 homomers, it could potentially explain the inward rectification as well. However, the changes in single channel properties cannot account for the increase in mEPSC amplitude or the inward rectification of AMPARs in the GluR1-S845A mutants, which should display a smaller GluR1-mediated current.

Our finding that S845 phosphorylation is necessary, but not sufficient, for multiplicative scaling, is consistent with a view on AMPAR trafficking proposing the existence of “slots” or “placeholders” to stabilize the receptors at synapses [43]. One possible explanation is that multiplicative scaling requires a two-step process: (1) phosphorylation of GluR1 at S845, which functionally up-regulates CP-AMPARs at synapses, either by mobilizing perisynaptic receptors or by increasing the functionality of existing synaptic receptors, and (2) generation of “slots” that stabilizes CP-AMPARs at the PSD in a multiplicative manner. Under conditions where S845 phosphorylation is increased without the generation of “slots”, as in the GluR1-S831A mutants and with isoproterenol treatment, synaptic scaling does not occur in a multiplicative manner. Interestingly, activating PKA signaling via isoproterenol up-regulates cell surface AMPAR expression, which is not mimicked in the S831A mutants with hyperphosphorylated S845. This suggests that PKA signaling may recruit additional mechanisms besides S845 phosphorylation to traffic AMPARs to the plasma membrane. An unexpected observation is that GluR1-S831A mutants paradoxically scaled down their mEPSCs when dark-exposed, which is opposite to scaling up of mEPSCs seen in wildtypes. This abnormal scaling was associated with a reduction in inward rectification indicative of losing functional CP-AMPARs from synapses. Because GluR1-S845 phosphorylation still increased in the dark-exposed GluR1-S831A mutants, it suggests that the S831 site probably plays an additional role to keep the CP-AMPARs at synapses. However, the exact role of GluR1-S831 needs to be fully elucidated.

While our study showed that GluR1-S845 phosphorylation is critical for visual deprivation-induced homeostatic synaptic plasticity, prior work on LTP and LTD outlined a role for S845 phosphorylation in Hebbian synaptic plasticity. Initially, it was shown that dephosphorylation of GluR1 at this site is associated with LTD in CA1 of the hippocampus [27], [44], [45]. Subsequent studies highlighted the importance of S845 phosphorylation for LTP in the CA1 region [22], [46], likely by “priming” GluR1 containing AMPARs for synaptic insertion [47], [48]. However, mutating the GluR1-S845 phosphorylation site does not block LTP [32]. In contrast to these studies in the hippocampus, the GluR1-S845 site is necessary for both LTP and LTD in layer 2/3 of the visual cortex [28] consistent with the PKA dependence of synaptic plasticity in these layers [49], [50], [51], [52] (but see [53]). These reports emphasize that the reversible regulation of phosphorylation at S845 is an important player in bidirectional Hebbian synapse-specific plasticity, and may provide a mechanistic basis for neuromodulation of LTP and LTD in the visual cortex [28], [54]. Our study demonstrates that similar mechanisms are recruited for homeostatic regulation of synaptic AMPARs by visual deprivation. This implies that despite differences in the induction mechanisms, both synapse-specific plasticity and homeostatic synaptic scaling may be mediated by a common downstream molecular event. It is of interest that GluR1-S845 is a downstream target of various neuromodulatory systems coupled to the PKA signaling pathway. Activation of Gs-coupled receptors like β-adrenergic receptor and D1/D5 receptors readily increases phosphorylation of GluR1 at S845 [55], [56], [57], [58], [59]. In addition, there is evidence that S845 phosphorylation by β-adrenergic receptor agonists “primes” AMPARs for LTP [28], [47], [60]. Whether the dark-exposure induced increase in S845 phosphorylation and synaptic CP-AMPARs will aid in subsequent LTP expression remains to be examined. These molecular events may provide an alternative explanation for previous studies showing larger LTP in layer 2/3 of the primary visual cortex following dark-rearing from birth [61], [62]. In any event, if experience-induced Hebbian and homeostatic synaptic plasticity share similar downstream events, it is predicted that they will influence each other's expression.

## Materials and Methods

### Dark-exposing animals

All animal procedures followed the National Institutes of Health (NIH) guidelines, and were approved by the University of Maryland Institutional Animal Care and Use Committee (IACUC, Protocol# R0978, approval date 10/12/2009). Wildtype (WT) and homozygous mice from GluR1-S831A and GluR1-S845A gene knockin lines (genetic background: C57BL6) [28], [32] were raised in a normal lighted environment (12 hr light/12 hr dark cycle) until postnatal age 21 days (P21). DE was initiated at P21 for 2 days, while control (NR) animals were continuously raised in the normal lighted condition for the same duration. The animals in the dark were cared for using infrared vision goggles under dim infrared light. After DE, some of the mice were taken out to the lighted environment for 1 day to study the effect of re-exposure to light (D+L).

### Whole-cell recording

Visual cortex slices were prepared as previously described [17], [18]. In brief, mice were decapitated under deep isoflurane anesthesia, and visual cortex was quickly dissected and sectioned (300 µm thickness). After ≥1 hr of recovery, a slice was moved to a submersion-type recording chamber mounted on a stage of an upright microscope (E600 FN, Nikon) equipped with infrared oblique illumination. Layer 2/3 pyramidal cells were visually identified and patched using a whole-cell patch pipette (tip resistance: 3–5 MΩ) filled with intracellular solution (in mM: 130 Cs-gluconate, 8 KCl, 1 EGTA, 10 HEPES, 4 ATP and 5 QX-314; pH 7.4; 285–295 mOsm).

AMPAR-mediated mEPSCs were recorded as previously described [18]. In brief, 1 µM TTX, 20 µM bicuculline, and 100 µM D,L-APV were added to the ACSF (in mM: 124 NaCl, 5 KCl, 1.25 NaH_2_PO_4_, 26 NaHCO_3_, 10 glucose, 1 MgCl_2_, 2 CaCl_2_; saturated with 95% O_2_/5% CO_2_, 2 ml/min, 30±1°C) to isolate mEPSCs. mEPSCs were recorded at a holding potential (Vh) of −80 mV using Axopatch-clamp amplifier (Axon Instruments), acquired using the Igor Pro™ software (Wave Metrics), and analyzed using the Mini Analysis Program (Synaptosoft). The detection threshold was set at 3 times the Root Mean Square (RMS) noise, and there was no significant difference in RMS noise across the experimental groups (data not shown). Cells showing dendritic filtering, as assessed by a negative correlation between mEPSC amplitude and rise time, were excluded from analysis, as well as mEPSCs with greater than 3 msec rise time. Average mEPSC amplitude and frequency were calculated and compared across different experimental groups using one-factor ANOVA or unpaired Student's t-test.

Evoked AMPAR-mediated EPSCs were measured from layer 2/3 pyramidal cells in response to stimulation through a bipolar electrode placed in layer 4. To isolate the AMPAR component, 100 µM D,L-APV and 40 µM bicuculline were added to the ACSF. The concentration of CaCl_2_ and MgCl_2_ in the ACSF were changed to 4 mM and 2 mM, respectively, to prevent polysynaptic responses upon stimulation in the presence of bicuculline. Intracellular recording solution containing 200 µM spermine (in mM: 90 CsMeSO_3_H, 5 MgCl_2_, 8 NaCl, 10 EGTA, 20 HEPES, 1 QX-314, 0.5 Na_3_GTP, and 2 Mg•ATP, pH 7.2, 250–270 mOsm) was used. For generating I-V curves for rectification measurements, cells were held at −60, −40, −20, 0, +20 and +40 mV. Inward rectification (IR) index was calculated by dividing the absolute amplitude of average EPSC measured at −60 mV by that at +40 mV. There were no significant differences in reversal potentials, calculated using equations generated by fitting a linear regression curve to the current values collected at negative holding potentials, between groups (data not shown). Only the cells and recording conditions that met the following criteria were studied: Vm at break-in ≤−65 mV, input R ≥200 MΩ, series R≤25 MΩ. Cells were discarded if input R or series R changed more than 15%. Junction potentials were typically ≤10 mV, and were left uncompensated.

### Postsynaptic density (PSD) preparation

Visual cortices were gently homogenized on ice in HEPES-buffered sucrose (0.32 M sucrose, 4 mM HEPES, pH 7.4) containing 2 mM EGTA, 50 mM NaF, 10 mM sodium pyrophosphate, 1 mM sodium orthovanadate, 1 µM okadaic acid, and protease inhibitors (Protease Inhibitor Cocktail, Pierce). Primary visual cortices from two animals were pooled together to generate one data point. The homogenates (H) were centrifuged at 800× g for 10 min (4°C) to remove pelleted nuclear fraction (P1), and the resulting supernatants (S1) were centrifuged at 10,000× g for 15 min (4°C) to yield the crude membrane pellets (P2). P2 fractions were resuspended in HEPES-buffered sucrose with inhibitors and respun at 10,000× g for 15 min (4°C) to yield the washed crude membrane fractions (P2′). P2′ fractions were lysed by hypo-osmotic shock in ice-cold 4 mM HEPES (pH 7.4, with inhibitors), and centrifuged at 25,000× g for 20 min to generate lysed synaptosomal membrane fractions (P3). P3 was subsequently resuspended in HEPES-buffered sucrose with inhibitors, and run on a discontinuous sucrose gradient (1.2 M, 1.0 M, and 0.8 M sucrose with inhibitors) at 150,000× g for 2 hours (4°C). Synaptic plasma membrane (SPM) fractions were collected between 1.0 M and 1.2 M sucrose and diluted with 2.5 volumes of 4 mM HEPES with inhibitors. SPM was pelleted by centrifugation at 150,000× g for 30 min (4°C), resuspended in 0.5% Triton X-100, HEPES-EDTA solution (50 mM HEPES, 2 mM EDTA, pH 7.4) with inhibitors, and rotated for 15 min at 4°C. Solubilized SPM was then centrifuged at 32,000× g for 20 min to pellet the postsynaptic density fraction (PSD). PSD fractions were resuspended in gel sample buffer and processed for SDS-PAGE (4 µg of PSD protein per lane) and immunoblot analysis using GluR1 (sc-55509, Santa Cruz), GluR2 (AB1768, Chemicon/Millipore), and GluR3 (MAB5416, Chemicon/Millipore) antibodies.

### Steady-state surface biotinylation

Visual cortex slices (400 µm thick) were prepared as described above. After 30 min recovery at room temperature, the slices were transferred to 30°C for additional 30 min recovery. The slices were then transferred to ice-cold ACSF for 10 min, and subsequently to ice-cold ACSF containing 2 mg/ml biotin (EZ-Link Sulfo-NHS-Biotin, Pierce) saturated with 5% CO_2_/95% O_2_ for 15 min. The slices were then washed in tris-buffered saline (TBS: 50 mM Tris, 0.9% NaCl, pH 7.4) containing 100 mM glycine 5 times 1 min each before homogenized in ice-cold 0.2% SDS/1% Triton X-100 IPB (20 mM Na_3_PO_4_, 150 mM NaCl, 10 mM EDTA, 10 mM EGTA, 10 mM Na_4_P_2_O_7_, 50 mM NaF, and 1 mM Na_3_VO_4_, pH 7.4; with 1 µM okadaic acid and 10 KIU/ml aprotinin) by ∼30 gentle strokes using glass-teflon tissue homogenizers (Pyrex). The homogenates were centrifuged for 10 min at 13,200× g, 4°C. Protein concentration of the supernatant was measured and normalized to 2 or 4 mg/ml. Some of the supernatants were saved as inputs by adding gel sampling buffer and boiled for 5 min. 300 µg of each supernatant was mixed with neutravidin slurry [1∶1 in 1% Triton X-100 IPB (TX-IPB)] and rotated overnight at 4°C. The neutravidin beads were isolated by brief centrifugation at 1,000× g. Some of the supernatants were saved by adding gel sample buffer and boiled for 5 min. The neutravidin beads were washed 3 times with 1% TX-IPB, 3 times with 1% TX-IPB containing 500 mM NaCl, followed by 2 washes in 1% TX-IPB. The biotinylated surface proteins were then eluded from the neutravidin beads by boiling in gel sampling buffer for 5 min. The input (total homogenate), supernatant (intracellular fraction), and biotinylated samples (surface fraction) were run on the same gel, and processed for immunoblot analysis using GluR1 (sc-55509, Santa Cruz), GluR2 (AB1768, Chemicon/Millipore), and actin (MAB1501, Chemicon/Millipore) antibodies. The band intensity in the input lanes and biotin lanes, which fell within the linear range, was quantified to calculate the % of total GluR1 or GluR2 on the surface for each sample.

### Immunoblot analysis

SDS-PAGE gels were transferred to polyvinyl difluoride (PVDF) membranes (Immobilon™, Millipore). The PVDF membrane blots were blocked for ∼1 hr in blocking buffer (1% bovine serum albumin and 0.1% Tween-20 in phosphate buffered saline (PBS), pH 7.4), and subsequently incubated for 1–2 hrs in primary antibodies (Ab's) diluted in blocking buffer. After 5 times 5 min washes in blocking buffer, the blots were incubated for 1 hr in 2^nd^ Ab linked to alkaline phosphatase (AP) diluted 1∶10,000 in blocking buffer. The blots were washed 5 times (5 min each), and developed using enhanced chemifluorescence substrate (ECF substrate, Amersham). The ECF blots were scanned using a Versa Doc 3000™ gel imaging system (Bio-Rad), and quantified using Quantity One software (Bio-Rad). The signal of each sample on a blot was normalized to the average signal from samples of NR (for NR and DR comparison) or WT (for WT and mutant comparison) group to obtain the % of average NR or % of average WT values, which were compared across different experimental groups using unpaired Student's t-test.

For analysis of phosphorylation, ECLplex (GE Health) system was used. In brief, the blots were incubated simultaneously in phospho-specific Ab for GluR1-S831 (rabbit polyclonal, affinity purified in-house) or GluR1-S845 (ab3901, Abcam) and GluR1-C terminal Ab (sc-55509, Santa Cruz). After washes in blocking buffer, the blots were incubated simultaneously in 2^nd^ Abs linked to Cy3 and Cy5. After washes, blots were scanned using Typhoon Trio (GE Health), and signals were quantified using Image Quant TL software (GE Health). Signal from phospho-specific Ab was divided by signal from GluR1-C terminal Ab to obtain the fraction of phosphorylated GluR1 in each sample. This value was then normalized to the average value from NR or WT samples respectively to obtain the % of average NR or % of average WT values, which were compared across different experimental groups using one-factor ANOVA or unpaired Student's t-test. The biotinylation blots were also probed simultaneously with GluR1 and GluR2 antibodies using the ECL plex system, and subsequently reprobed for actin.

## Supporting Information

Figure S1 **Explanation of multiplicative scaling.** Initial strengths across different synapses are not likely identical due to synapse-specific plasticity mechanisms such as LTP and LTD. The initial strengths of individual synapses are designated as *a_1_*, *a_2_*, *a_3_*, through *a_x_*, such that the average synaptic strength is *A*. When these synapses scale multiplicatively, by multiplying a scaling factor of *f* to individual synaptic strengths, the relative differences in the strength of each synapse is preserved even when the average strength of synaptic transmission is changed to *Af*.
(TIF)

Figure S2 **Inward rectification depends on intracellular spermine.** The inward rectification of evoked AMPAR-EPSC in normal-reared GluR1-S845A mutants **(A)** and GluR1-S831A mutants **(B)** depended on the presence of intracellular polyamines. Left: comparison of the inward rectification index measured with (+Sp, white) or without (–Sp, light blue) spermine in the internal solution. Note that without spermine, the inward rectification index is reduced similar to normal-reared wildtype values (see Fig. 1c). Inward rectification index (I_–60 mV_/I_+40 mV_): S845A +Sp = 3.6±0.4, n = 10; S845A –Sp = 1.7±0.2, n = 4; S831A +Sp = 4.7±0.4, n = 9; S831A –Sp = 1.9±0.2, n = 3. *: p<0.001, t-test. Right: superimposed example traces taken at −60 mV and +40 mV for each group.
(TIF)

Table S1 **Comparison of mEPSC kinetics and neuronal properties.**
(DOC)
