# Supplementary figures and images for: Phosphorylation of AMPA Receptors Is Required for Sensory Deprivation-Induced Homeostatic Synaptic Plasticity
Source: PLoS One. 2011 Mar 31;6(3):e18264. doi: 10.1371/journal.pone.0018264 (PMC3069067; doi:10.1371/journal.pone.0018264)

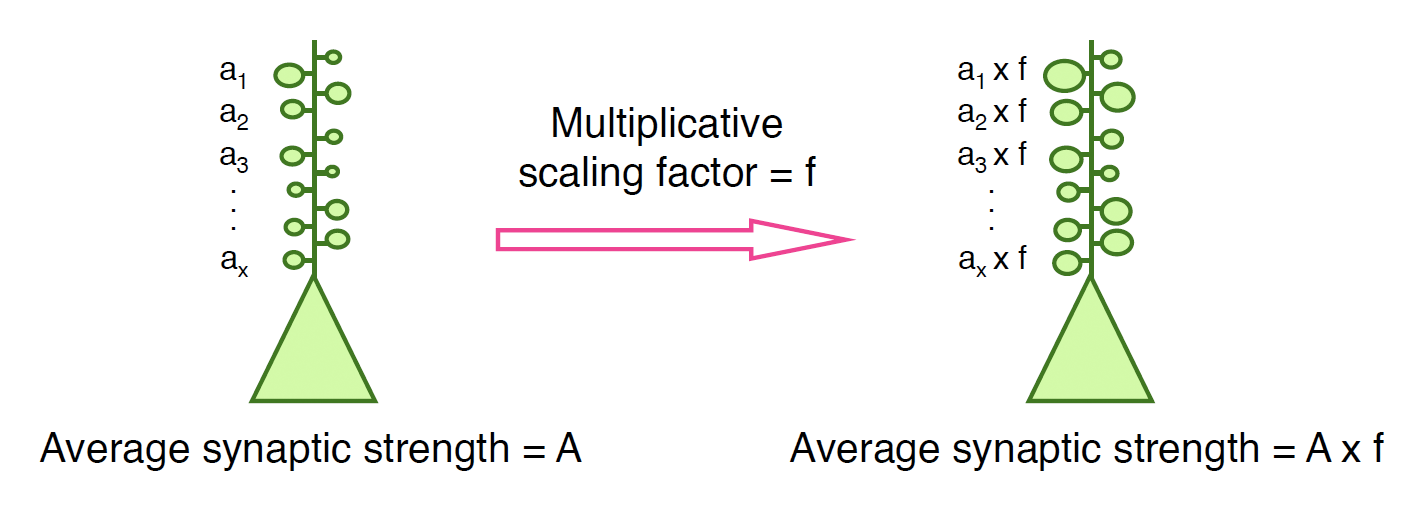

Supplement: Figure S1 — Explanation of multiplicative scaling. Initial strengths across different synapses are not likely identical due to synapse-specific plasticity mechanisms such as LTP and LTD. The initial strengths of individual synapses are designated as a1, a2, a3, through ax, such that the average synaptic strength is A. When these synapses scale multiplicatively, by multiplying a scaling factor of f to individual synaptic strengths, the relative differences in the strength of each synapse is preserved even when the average strength of synaptic transmission is changed to Af. (TIF) [file pone.0018264.s001.tif]

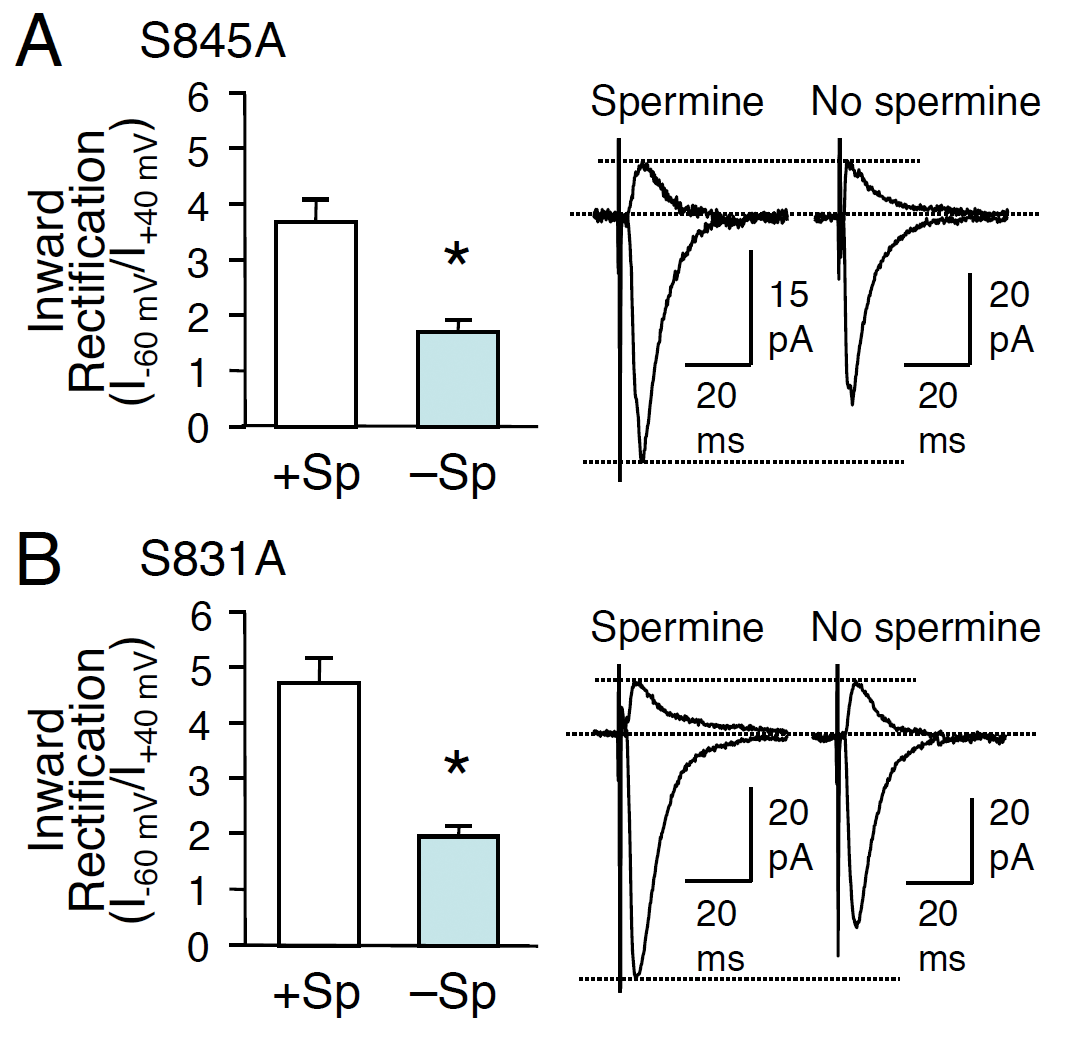

Supplement: Figure S2 — Inward rectification depends on intracellular spermine. The inward rectification of evoked AMPAR-EPSC in normal-reared GluR1-S845A mutants (A) and GluR1-S831A mutants (B) depended on the presence of intracellular polyamines. Left: comparison of the inward rectification index measured with (+Sp, white) or without (–Sp, light blue) spermine in the internal solution. Note that without spermine, the inward rectification index is reduced similar to normal-reared wildtype values (see Fig. 1c). Inward rectification index (I–60 mV/I+40 mV): S845A +Sp = 3.6±0.4, n = 10; S845A –Sp = 1.7±0.2, n = 4; S831A +Sp = 4.7±0.4, n = 9; S831A –Sp = 1.9±0.2, n = 3. *: p<0.001, t-test. Right: superimposed example traces taken at −60 mV and +40 mV for each group. (TIF) [file pone.0018264.s002.tif]
