# Supplementary material for: Phosphorylation of AMPA Receptors Is Required for Sensory Deprivation-Induced Homeostatic Synaptic Plasticity
Source: PLoS One. 2011 Mar 31;6(3):e18264. doi: 10.1371/journal.pone.0018264 (PMC3069067; doi:10.1371/journal.pone.0018264)
Supplement: Table S1 — Comparison of mEPSC kinetics and neuronal properties. (DOC) [file pone.0018264.s003.doc]

**Table S1. Comparison of mEPSC kinetics and n**euronal properties.

|  | Conditions | mEPSC  rise time  (ms) | mEPSC  decay time constant (τ) (ms) | R_in_ (MΩ) | R_ser_ (MΩ) |
| --- | --- | --- | --- | --- | --- |
| WT | NR  (n = 19) | 1.6 ± 0.05 | 4.7 ± 0.2 | 413 ± 48 | 19 ± 0.4 |
|  | DE  (n = 8) | 1.5 ± 0.03 | 3.5 ± 0.2* | 336 ± 24 | 18 ± 0.7 |
| S831 WT | NR  (n = 8) | 1.7 ± 0.09 | 4.8 ± 0.1 | 323 ± 14 | 19 ± 0.7 |
|  | NR + Iso  (n = 9) | 1.5 ± 0.06 | 3.5 ± 0.2^#^ | 388 ± 54 | 20 ± 0.4 |
| S831A | NR  (n = 11) | 1.5± 0.06 | 4.0 ± 0.2^#^ | 383 ± 47 | 19 ± 0.8 |
|  | DE  (n = 10) | 1.5 ± 0.07 | 4.8 ± 0.4 | 368 ± 32 | 21 ± 0.8 |
|  | D+L  (n = 9) | 1.6 ± 0.09 | 4.3 ± 0.2 | 418 ± 62 | 19 ± 1.1 |
| S845 WT | NR  (n = 11) | 1.6 ± 0.06 | 4.5 ± 0.3 | 486 ± 81 | 19 ± 0.5 |
| S845A | NR  (n = 10) | 1.7± 0.10 | 4.7 ± 0.3 | 303 ± 25 | 20 ± 0.8 |
|  | DE  (n = 11) | 1.5 ± 0.08 | 4.1 ± 0.2 | 449 ± 81 | 18 ± 1.1 |
|  | D+L  (n = 12) | 1.5 ± 0.05 | 4.5 ± 0.3 | 383 ± 33 | 19 ± 0.7 |

* denotes statistically significant difference from NR condition using t-test with p < 0.001.

# denotes statistically different from normal-reared WT of S831A line (S831 WT-NR) at p < 0.02 using the Fisher’s PLSD posthoc analysis following a one-factor ANOVA [F(2,25) = 3.719, p < 0.01].
